# Deficiency of ADAMTS-13 in pediatric patients with severe sepsis and impact on in-hospital mortality

**DOI:** 10.1186/1471-2431-13-44

**Published:** 2013-03-28

**Authors:** Farheen Karim, Salman Naseem Adil, Bushra Afaq, Anwar ul Haq

**Affiliations:** 1 Section of Hematology, Department of Pathology and Microbiology, The Aga Khan University Hospital, Stadium Road, Karachi, 74800, Pakistan; 2 Department of Paediatrics, The Aga Khan University Hospital, Karachi, Pakistan

**Keywords:** ADAMTS-13, Sepsis, Deficiency, Pediatric

## Abstract

**Background:**

The enzyme involved in regulating the size of vWF (von Willebrand factor) in plasma is ADAMTS-13 (A disintegrin and metalloprotease with thrombospondin type-1 motives). Deficient proteolysis of ULvWF (ultra large von Willebrand factor) due to reduced ADAMTS-13 activity results in disseminated platelet-rich thrombi in the microcirculation characteristic of thrombotic thrombocytopenic purpura. Reduced ADAMTS-13 has also been observed in severe sepsis and is associated with poor survival. We conducted this study to detect ADAMTS-13 deficiency and its impact on in-hospital mortality in pediatric patients with severe sepsis.

**Methods:**

Pediatric patients diagnosed with severe sepsis were recruited for the study. Baseline clinical characteristics were noted. ADAMTS-13 antigen levels were assayed by ELISA. According to ADAMTS-13 levels, patients were grouped as deficient and non-deficient. Comparison was done with regard to some clinical and biological characteristics and in-hospital mortality between the two groups.

**Results:**

A total of 80 patients were enrolled in the study. The median age of the patients was 3.1 years (Range: 0.1-15 years). ADAMTS-13 deficiency with levels less than 350 ng/dl was found in 65% patients. In patients with ADAMTS-13 deficiency, 75.6% had low platelets of less than 150 × 10^9^/L. In-hospital mortality was 42.3% and 35.7% in ADAMTS-13 deficient and non-deficient group, respectively.

**Conclusion:**

Majority of the pediatric patients admitted to hospital with severe sepsis exhibit ADAMTS-13 deficiency. ADAMTS-13 deficiency might play a role in sepsis-induced thrombocytopenia. More studies are needed to evaluate the role of ADAMTS-13 deficiency on in-hospital mortality.

## Background

von Willebrand factor (vWF), a multimeric protein that mediates platelet adhesion and aggregation at sites of vascular injury is stored in specialized storage organelles known as Weibel-Palade bodies within the endothelial cells and in the alpha granules of platelets [1]. It is released from the stimulated endothelium as unusually large (UL) multimer [2,3]. ULvWF is the hyperactive form of von Willebrand factor (vWF), having more affinity for platelets favoring platelet aggregation and formation of microvascular thrombi [4,5]. This ULvWF relies upon an enzyme known as ADAMTS-13 (A disintegrin and metalloprotease with thrombospondin type-1 motives) for its cleavage and thus conversion into a less active form [6]. Deficient proteolysis of ULvWF due to reduced ADAMTS-13 activity results in disseminated platelet-rich thrombi in the microcirculation seen in thrombotic microangiopathies (TMA). Decreased levels of ADAMTS-13 are particularly seen in TTP. Low levels have also been observed in various other disease states including liver disease [7], malignancy [8], systemic lupus erythematosus [8], disseminated intravascular coagulation [9] and severe sepsis [10].

As elevated levels of vWF are observed in several inflammatory disease states including sepsis, it is believed that normal or mildly reduced levels of ADAMTS-13 activity may not be sufficient enough to control vWF multimer size [10]. The net result is an accumulation of extremely thrombogenic ULvWF on the surface of the disturbed endothelium, thus propagating pathological platelet–endothelial interactions, which in combination with other prothrombotic changes seen in sepsis could contribute to enhanced microvascular thrombosis, platelet consumption, disseminated intravascular coagulation and eventually multi-organ failure [11]. Reduction in ADAMTS levels to less than 10% of normal result in clinically apparent thrombosis and thrombocytopenia [12].

Many studies have evaluated the role of ADAMTS-13 in severe sepsis. In a study conducted in Japan, decreased ADAMTS-13 activity ( less than 5%) was found in 15.6% of patients with sepsis induced disseminated intravascular coagulation; whereas in USA a study showed that 31% of patients with severe sepsis had severe ADAMTS-13 deficiency [11,13]. It has also been reported that deficiency of ADAMTS-13 plays an important role in sepsis-associated thrombocytopenia, which is seen in more than 50% of patients with sepsis and correlates with poor prognosis in these patients [13]. Few studies have also shown that reduced ADAMTS-13 is associated with a poor survival rate in sepsis [14].

Sepsis is the leading cause of death in patients of all age groups presenting to the emergency of our hospital (23%; 95% CI 19-26%) [15] A worldwide systemic analysis carried out in 2008 indicated that Pakistan was among the five countries with very high pediatric mortality and sepsis accounted for 6% of all neonatal deaths [16]. Thus, the burden of sepsis and sepsis-related deaths is very high nationwide and preventive strategies are needed to decrease sepsis associated morbidity and mortality. Based on the background of association of ADAMTS-13 with sepsis, we conducted this study to detect ADAMTS-13 deficiency in pediatric patients with severe sepsis. Furthermore, we correlated ADAMTS-13 deficiency with sepsis associated thrombocytopenia and in-hospital mortality.

## Methods

### Study design and setting

The study was conducted in the section of Haematology, Department of Pathology and Microbiology, The Aga Khan University Hospital, Karachi. This was a cross-sectional study extending over one year from February 2010 to January 2011.

### Patient selection

80 pediatric patients admitted to The Aga Khan University Hospital and diagnosed as having severe sepsis according to the guidelines of the society of Critical Care Medicine Consensus Conference Committee were recruited for the study [17]. In short, selected patients met at least 3 out of the 4 systemic inflammatory response criteria (core temperature >38.5°C or <36°C; tachycardia >2 SD above normal for age; mean respiratory rate >2 SD above normal for age and leukocyte count elevated or depressed for age) in the presence of a known or a suspected infection proven by one or more of the following findings: bacteremia, purulent sputum; presence of clinical signs that are associated with high risk for infection such as cholangitis or peritonitis; detection of pathogenic microorganisms or white blood cells in a normally sterile body fluid such as urine or joint fluid and radiological evidence of pneumonia alongwith one of the following: cardiovascular organ dysfunction, acute respiratory distress syndrome or two or more organ dysfunctions (respiratory, renal, neurological, hematological or hepatic) [17].

Patients with a known thrombotic or bleeding disorder, patients receiving transfusion of blood products within a week of sample collection and those with end stage liver disease were excluded from the study.

### Consent

Written informed consent was obtained from the parent/guardian of the patient.

### Sample collection

Three milliliter of venous blood sample was taken from every patient into tubes containing 3.2% sodium citrate anticoagulant for analysis of ADAMTS-13 antigen levels. Platelet poor plasma was obtained after centrifugation of samples. Plasma samples were labeled and kept at −80°C until analysis.

### ADAMTS-13 analysis

ADAMTS-13 antigen levels were performed on platelet poor plasma sample collected in citrate by IMUBIND® ADAMTS-13 ELISA (American Diagnostica Inc, USA). ADAMTS-13 antigen level of less than 350 ng/dl was taken as deficient as defined by the manufacturer. Patients were categorized in two groups according to the ADAMTS-13 level as deficient and non-deficient. Comparison was done with regard to some clinical and biological characteristics and in-hospital mortality between the two groups.

### Statistical analysis

Data was entered and analyzed using SPSS (statistical package for social Sciences; Chicago Inc, USA) version 19.0. Statistical tests used for comparison of clinical characteristics included Mann–Whitney U test, Fisher exact test and Chi-square test depending on the scale of measurement. A *p-value* of <0.05 was considered statistically significant. Kaplan-Meier method was used for evaluating survival.

### Ethical review

The study was approved by the Ethical review committee of The Aga Khan University Hospital. (ERC approval Number: 1598-Path-ERC-2010).

## Results

A total of 80 patients were included in the study. The median age of the patients was 3.1 years (Range: 0.1-15 years). There were 45 (56.25%) males and 35 (43.75%) females. ADAMTS-13 deficiency with levels less than 350 ng/dl was found in 52 (65%) patients, whereas 28(35%) were not deficient. Microbiologically defined infection was present in 48 (60%) patients. The clinical characteristics were compared and do not appear to be different between the two groups (Table 1).

**Table 1:** Characteristics of patients in ADAMTS-13 deficient and non-deficient group

**Characteristics**	**ADAMTS-13 deficient group (n=52)**	**ADAMTS-13 non-deficient group (n=28)**	**p-value**
**Median age (years)**	3.1	3.2	-
**Gender [n(%)]**			
**Male**	31 (59.6%)	14 (50%)	0.408
**Female**	21 (40.4%)	14 (50%)	
**Mean hemoglobin(gm/dl)**	10.4	11.8	-
**Mean leucocyte count (× 10**^**9**^**/l)**	18.7	22	-
**Blood culture [n(%)]**			
**Positive**	35 (67.3%)	13 (46.4%)	0.069
**PT (sec)**	24	19.5	-
**APTT (sec)**	42	39	-

Overall, 37 (46.2%) patients were found to have platelets of less than 150 × 10^9^/L. Among those who had low platelets, 28 (75.6%) patients were in the ADAMTS-13 deficient group.

The mean duration of hospitalization was 12.4 days in patients with ADAMTS-13 deficiency, while it was 8.8 days in the non-deficient group. The difference was not statistically significant.

**Figure 1:**
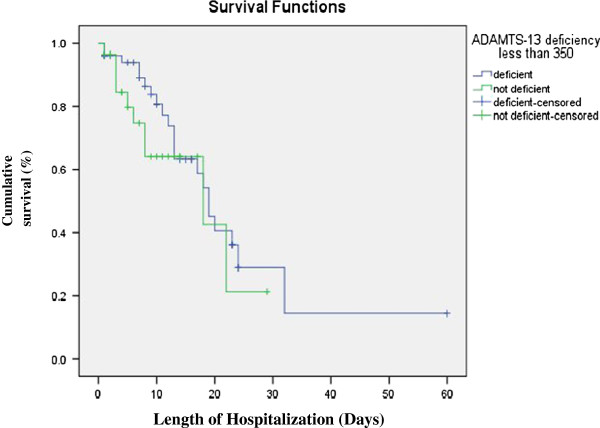
Cumulative survival of patients with and without ADAMTS-13 Deficiency.

In-hospital mortality was seen in thirty-two (40%) patients. Forty-two (52.5%) of the patients were discharged from the hospital in stable condition, while 6 (7.5%) were shifted to another hospital. Out of the total 32 patients who expired, 22 (42.3%) had ADAMTS-13 deficiency whereas 10 (35.7%) were not deficient. The cumulative survival of the patients between the two groups using Kaplan-Meire is shown in Figure 1. The difference was not statistically significant.

## Discussion

ADAMTS13 plays an important role in the processing of vWF. Primary ADAMTS13 deficiency caused by defects in the ADAMTS13 gene has been shown to cause Thrombotic thrombocytopenic purpura (TTP) [18]. TTP is a fatal thrombotic microangiopathic disease if emergency plasma pheresis is not started [19]. This shows the importance of the physiologic role of ADAMTS13-catalyzed cleavage of the unusually large vWF multimers in humans. While looking into the role of ADAMTS13 in common diseases associated with thrombotic microangiopathies, it was found that severe secondary ADAMTS13 deficiency is also present in patients with sepsis, connective tissue diseases, liver cirrhosis and acute inflammation [7,20].

Severe sepsis and septic shock are the consequence of an uncontrolled and extensive inflammatory response to infection which is associated with systemic activation of a number of host defense mechanisms [21]. Decreased ADAMTS-13 in sepsis has been suggested to be due to several potential mechanisms. These mechanisms include consumption of ADAMTS-13 due to on-going cleavage of excessively secreted ULvWF from activated endothelium during sepsis; inhibition of ADAMTS-13 by inflammatory cytokines such as interleukin −6 (IL-6) ; cleavage of ADAMTS-13 by proteases released from neutrophils during inflammatory conditions and last but not the least functional inactivation by coagulation proteinases [11,22]. Deficiency of ADAMTS-13 results in microthrombi in the circulation resulting in ischemia and end-organ damage.

Decreased levels have been observed in healthy volunteers following endotoxin infusion [23], in inflammatory diseases, and also in thrombocytopenia associated with severe sepsis [8,11,24]. Our results revealed that pediatric patients with severe sepsis had a high incidence of ADAMTS-13 deficiency. In our study population 65% of the patients were found to be deficient in ADAMTS-13. Our results match with the results reported by Scully et al. who also reported a high frequency of ADAMTS-13 deficiency in 48 children admitted to neonatal or pediatric intensive care unit. In this study 64% of the children showed reduced ADAMTS-13 levels [25]. Moore et al. also reported in his study a similar frequency of ADAMTS-13 deficiency seen in 60% patients suffering from disseminated intravascular coagulation [26].

Our results revealed that majority of the septic patients with thrombocytopenia were in the ADAMTS-13 deficient group. A study from USA by Nguyen et al., conducted on 21 pediatric patients with severe sepsis also concluded that ADAMTS-13 deficiency was associated with thrombocytopenia [13]. It is hypothesized that sepsis-associated thrombocytopenia is a result of platelet consumption due to aggregation of platelets in the microcirculation and thrombotic microangiopathy due to incomplete cleavage of ULVWF multimers as a consequence of ADAMTS-13 deficiency. Sepsis-associated thrombocytopenia has been correlated with poor prognosis [27].

Studies have shown that patients with low ADAMTS-13 activity have a poor survival. Hyun J et al. reported poor survival of patients with low ADAMTS-13 activity than patients with higher activity (p-value 0.0477) [14]. One of the studies showed that ADAMTS-13 activity was significantly lower in patients with severe sepsis and patients above the median of ADAMTS-13 activity presented a higher survival compared with those below the median activity [15]. In our study, survival of patients with and without ADAMTS-13 deficiency was not statistically significant. Few patients in our study (7.5%) were either shifted to another hospital due to unavailability of intensive care unit bed or left against medical advice. All of them were ADAMTS-13 deficient and were unstable. Since, we do not know about the mortality in these patients, they were not included in the analysis. Inclusion of these patients in analysis may have influenced the results. Furthermore, because of early recognition and improved care of patients with sepsis, the survival may have improved in those who were ADAMTS-13 deficient. Further studies are needed to better understand the effect of ADAMTS-13 deficiency on survival of patients.

The study has certain limitations like ADAMTS-13 was only assayed at the time of diagnosis and serial monitoring was not done so it could not be assessed whether or not the levels improved with clinical improvement of the patient.

We conclude that majority of the patients with severe sepsis have ADAMTS-13 deficiency that might also play a role in sepsis-induced thrombocytopenia. It shows that low ADAMTS-13 does not have a sole diagnostic value for TTP only but is also low in other disease states like sepsis. Both TTP and sepsis are associated with thrombotic microangiopathy and severe ADAMTS-13 deficiency. This raises the possibility of novel therapeutic strategies for patients with sepsis including plasma infusion; ADAMTS-13 supplementation and synthetic granulocyte elastase inhibitors. Synthetic granulocyte elastase inhibitors inhibit granulocyte elastase, an enzyme implicated in causing ADAMTS-13 deficiency in patients with sepsis. Analysis of ADAMTS-13 in sepsis would offer help in evaluating the status of the patient. Therefore, ADAMTS-13 may be used as a diagnostic marker in patients with sepsis.

## Conclusion

Majority of the pediatric patients admitted to hospital with severe sepsis exhibit ADAMTS-13 deficiency. ADAMTS-13 deficiency might play a role in sepsis-induced thrombocytopenia. More studies are needed to evaluate the role of ADAMTS-13 deficiency on in-hospital mortality.

## Abbreviations

vWF: von willebrand factor; ULvWF: Ultra large von Willebrand factor; TTP: Thrombotic thrombocytopenic purpura; PT: Prothrombin time; APTT: Activated partial thromboplastin time; SD: Standard deviation.

## Competing interest

The authors declare that they have no competing interests.

## Authors’ contributions

FK collected data, analyzed data and wrote the original manuscript. SNA critically analyzed the manuscript and provided guidance. BA performed all laboratory work. AH was the primary physician who helped in recruiting patients and data collection and analyzed the manuscript. All authors read and approved the final manuscript.

## Pre-publication history

The pre-publication history for this paper can be accessed here:

http://www.biomedcentral.com/1471-2431/13/44/prepub
